# Supplementary material for: Changes in the Quality Attributes of Selected Long-Life Food at Four Different Temperatures over Prolonged Storage
Source: Foods. 2022 Jul 6;11(14):2004. doi: 10.3390/foods11142004 (PMC9319022; doi:10.3390/foods11142004)
Supplement: Supplementary file 1 [file foods-11-02004-s001.zip › foods-1807743-supplementary.pdf]

Supplementary file

Table S1. Results of sensory analysis (appearance, consistency, flavour and off-flavour) of the instant goulash soup stored during a 24-month period at four different temperatures (-18°C; 5°C; 25°C and 40°C). The results are expressed as median (n = 24). *

Storage time (months)	Storage temperature (°C)	Appearance **	Consistency **	Flavour **	Off-flavour ***
0	-	2 A	2 A	2 A	1 A
1	40	5 B	5 B	3 B	2 B
3	-18	2^{a}A	2^{a}A	2^{a}A	1^{a}A
	5	2^{a}A	4^{b}B	2^{a}A	1^{a}A
	25	2^{a}A	4^{b}B	2^{a}A	1^{a}A
	40	5^{b}B	6^{c}B	5^{b}B	5^{b}B
6	5	3^{a}B	4^{a}B	2^{a}A	1^{a}A
	25	3^{a}B	4^{a}B	3^{b}B	2^{b}B
	40	5^{b}B	6^{b}B	6cB	6^{c}B
9	5	3^{a}B	4^{a}B	3^{a}B	2^{a}B
	25	4^{b}C	4^{a}B	3^{a}B	2^{a}B
	40	5^{c}B	6^{b}B	6^{b}B	6^{b}B
12	-18	4^{a}B	4^{a}B	4^{a}B	2^{a}B
	5	4^{a}C	4^{b}B	5^{b}C	2^{a}B
	25	4^{a}C	4^{b}B	3^{c}B	3^{b}C
	40	NE ****	NE	NE	NE
15	5	4^{a}C	4^{a}B	5^{a}B	3^{a}C
	25	4^{a}C	4^{a}B	5^{a}C	5^{b}D
	40	NE	NE	NE	NE
18	5	4^{a}C	4^{a}B	5^{a}B	3^{a}C
	25	4^{a}C	4^{a}B	7^{b}D	7^{b}E
	40	NE	NE	NE	NE
21	5	5 D	5 C	5 B	3 C
	25	NE	NE	NE	NE
	40	NE	NE	NE	NE
24	-18	4^{a}B	5^{a}C	7^{a}C	7^{a}C
	5	5^{b}D	5^{a}C	5^{b}B	3^{b}C
	25	NE	NE	NE	NE
	40	NE	NE	NE	NE

* The means within a column (the difference between the storage temperature) followed by different superscript letters differ ($P < 0.05$). The means within a column (the difference between the storage period) followed by different capital letters differ ($P < 0.05$).

** Seven-point scales (1-excellent, 4-good, 7-unacceptable) were used.

*** A seven-point scale (1-negligible, 4-medium, 7-excessive) was used.

**** NE – not evaluated due to the results of sensory analysis and/or microbiological analysis.

Table S2. Results of sensory analysis (appearance, consistency, flavour and off-flavour) of the Szeged goulash stored during a 24-month period at four different temperatures (-18°C; 5°C; 25°C and 40°C). The results are expressed as median (n = 24). *

Storage time (months)	Storage temperature (°C)	Appearance **	Consistency **	Flavour **	Off-flavour ***
0	-	2 A	2 A	2 A	1 A
1	40	5 B	3 B	4 B	5 B
3	-18	2^{a}A	2^{a}A	3^{a}B	1^{a}A
	5	2^{a}A	2^{a}A	2^{b}A	1^{a}A
	25	2^{a}A	2^{a}A	2^{b}A	1^{a}A
	40	5^{b}B	3^{b}B	4^{c}B	5^{b}B
6	5	3^{a}B	2^{a}A	2^{a}A	1^{a}A
	25	3^{a}B	2^{a}A	2^{a}A	1^{a}A
	40	5^{b}B	3^{b}B	4^{b}B	5^{b}B
9	5	3^{a}B	2^{a}A	2^{a}A	1^{a}A
	25	4^{b}C	2^{a}A	2^{a}A	1^{a}A
	40	5^{c}B	4^{b}C	4^{b}B	5^{b}B
12	-18	4^{a}B	2^{a}A	4^{a}C	2^{a}B
	5	4^{a}C	2^{a}A	3^{b}B	1^{b}A
	25	4^{a}C	2^{a}A	2^{c}A	1^{b}A
	40	5^{b}B	4^{b}C	4^{a}B	5^{c}B
15	5	4^{a}C	2^{a}A	3^{a}B	2^{a}B
	25	4^{a}C	2^{a}A	3^{a}B	2^{a}B
	40	6^{b}C	5^{b}D	5^{b}C	5^{b}B
18	5	4^{a}C	2^{a}A	4^{a}C	3^{a}C
	25	4^{a}C	2^{a}A	3^{b}B	4^{b}C
	40	6^{b}C	5^{b}D	5^{c}C	5^{b}B
21	5	5^{a}D	2^{a}A	5^{a}D	6^{a}D
	25	5^{a}D	2^{a}A	5^{a}C	6^{a}D
	40	6^{b}C	6^{b}E	6^{b}D	6^{a}C
24	-18	4^{a}B	2^{a}A	4^{a}C	3^{a}C
	5	5^{b}D	4^{b}B	6^{b}E	6^{b}D
	25	6^{c}E	5^{c}B	6^{b}D	7^{c}E
	40	6^{c}C	7^{d}E	7^{c}E	7^{c}D

* The means within a column (the difference between the storage temperature) followed by different superscript letters differ ($P < 0.05$). The means within a column (the difference between the storage period) followed by different capital letters differ ($P < 0.05$).

** Seven-point scales (1-excellent, 4-good, 7-unacceptable) were used.

*** A seven-point scale (1-negligible, 4-medium, 7-excessive) was used.

Table S3. Results of sensory analysis (appearance, consistency, flavour and off-flavour) of the canned chicken meat stored during a 24-month period at four different temperatures (-18°C; 5°C; 25°C and 40°C). The results are expressed as median (n = 24). *

Storage time (months)	Storage temperature (°C)	Appearance **	Consistency **	Flavour **	Off-flavour ***
0	-	1 A	1 A	2 A	1 A
1	40	3 B	3 B	5 B	5 B
3	-18	1^{a}A	1^{a}A	3^{a}B	2^{a}B
	5	1^{a}A	1^{a}A	3^{a}B	2^{a}B
	25	1^{a}A	1^{a}A	4^{b}B	3^{b}B
	40	3^{b}B	3^{b}B	5^{c}B	5^{c}B
6	5	1^{a}A	1^{a}A	4^{a}C	4^{a}C
	25	1^{a}A	1^{a}A	4^{a}B	4^{a}C
	40	3^{b}B	3^{b}B	6^{b}C	6^{b}C
9	5	1^{a}A	1^{a}A	4^{a}C	4^{a}C
	25	1^{a}A	1^{a}A	5^{b}C	5^{b}D
	40	4^{b}C	4^{b}C	7^{c}D	7^{c}D
12	-18	1^{a}A	1^{a}A	4^{a}C	4^{a}C
	5	1^{a}A	1^{a}A	4^{a}C	4^{a}C
	25	1^{a}A	1^{a}A	5^{b}C	5^{b}D
	40	NE ****	NE	NE	NE
15	5	1^{a}A	1^{a}A	4^{a}C	4^{a}C
	25	1^{a}A	1^{a}A	5^{b}C	5^{b}D
	40	NE	NE	NE	NE
18	5	1^{a}A	1^{a}A	5^{a}D	5^{a}D
	25	2^{b}B	1^{a}A	5^{a}C	5^{a}D
	40	NE	NE	NE	NE
21	5	2^{a}B	1^{a}A	6^{a}E	5^{a}D
	25	3^{b}C	1^{a}A	7^{b}D	7^{b}E
	40	NE	NE	NE	NE
24	-18	2^{a}B	3^{a}B	6^{a}D	5^{a}A
	5	3^{b}B	2^{b}B	6^{b}E	6^{b}B
	25	NE	NE	NE	NE
	40	NE	NE	NE	NE

* The means within a column (the difference between the storage temperature) followed by different superscript letters differ ($P < 0.05$). The means within a column (the difference between the storage period) followed by different capital letters differ ($P < 0.05$).

** Seven-point scales (1-excellent, 4-good, 7-unacceptable) were used.

*** A seven-point scale (1-negligible, 4-medium, 7-excessive) was used.

**** NE – not evaluated due to the results of sensory analysis and/or microbiological analysis.

Table S4. Results of sensory analysis (appearance, consistency, flavour and off-flavour) of the pork pâté stored during a 24-month period at four different temperatures (-18°C; 5°C; 25°C and 40°C). The results are expressed as median (n = 24). *

Storage time (months)	Storage temperature (°C)	Appearance **	Consistency **	Flavour **	Off-flavour ***
0	-	3 A	3 A	3 A	1 A
1	40	4 B	3 A	3 A	1 A
3	-18	4^{a}B	3^{a}A	3^{a}A	1^{a}A
	5	4^{a}B	3^{a}A	3^{a}A	1^{a}A
	25	4^{a}B	3^{a}A	3^{a}A	1^{a}A
	40	4^{a}B	3^{a}A	4^{b}B	2^{b}B
6	5	4^{a}B	3^{a}A	3^{a}A	1^{a}A
	25	4^{a}B	3^{a}A	3^{a}A	1^{a}A
	40	4^{a}B	3^{a}A	4^{b}B	2^{b}B
9	5	4^{a}B	3^{a}A	3^{a}A	1^{a}A
	25	4^{a}B	3^{a}A	3^{a}A	2^{b}B
	40	4^{a}B	4^{b}B	5^{b}C	4^{c}C
12	-18	4^{a}B	3^{a}A	4^{a}B	1^{a}A
	5	4^{a}B	3^{a}A	3^{b}A	1^{a}A
	25	4^{a}B	3^{a}A	3^{b}A	2^{b}B
	40	5^{b}C	6^{b}C	5^{c}C	4^{c}C
15	5	4^{a}B	3^{a}A	3^{a}A	1^{a}A
	25	4^{a}B	3^{a}A	3^{a}A	2^{b}B
	40	5^{b}C	6^{b}C	5^{b}C	5^{c}D
18	5	4^{a}B	3^{a}A	3^{a}A	1^{a}A
	25	4^{a}B	3^{a}A	4^{a}B	3^{b}C
	40	5^{b}C	6^{b}C	5^{b}C	5^{c}D
21	5	4^{a}B	3^{a}A	4^{a}B	1^{a}A
	25	4^{a}B	3^{a}A	4^{a}B	4^{b}D
	40	5^{b}C	6^{b}C	6^{b}D	6^{c}E
24	-18	4^{a}B	3^{a}A	4^{a}B	1^{a}A
	5	4^{a}B	3^{a}A	4^{a}B	1^{a}A
	25	4^{a}B	3^{a}A	4^{a}B	4^{b}D
	40	5^{b}C	6^{b}C	7^{b}E	7^{c}F

* The means within a column (the difference between the storage temperature) followed by different superscript letters differ ($P < 0.05$). The means within a column (the difference between the storage period) followed by different capital letters differ ($P < 0.05$).

** Seven-point scales (1-excellent, 4-good, 7-unacceptable) were used.

*** A seven-point scale (1-negligible, 4-medium, 7-excessive) was used.

Table S5. Results of sensory analysis (appearance, consistency, flavour and off-flavour) of the canned tuna fish stored during a 24-month period at four different temperatures (-18°C; 5°C; 25°C and 40°C). The results are expressed as median (n = 24). *

Storage time (months)	Storage temperature (°C)	Appearance **	Consistency **	Flavour **	Off-flavour ***
0	-	2 A	2 A	2 A	1 A
1	40	3 B	3 B	3 B	2 B
3	-18	2^{a}A	3^{a}B	2^{a}A	1^{a}A
	5	2^{a}A	2^{b}A	2^{a}A	1^{a}A
	25	2^{a}A	2^{b}A	2^{a}A	1^{a}A
	40	3^{b}B	3^{b}B	3^{b}B	2^{b}B
6	5	2^{a}A	2^{a}A	2^{a}A	1^{a}A
	25	4^{b}B	2^{a}A	3^{b}B	2^{b}B
	40	3^{c}B	3^{b}B	3^{b}B	2^{b}B
9	5	3^{a}B	2^{a}A	3^{a}B	2^{a}B
	25	4^{b}B	2^{a}A	3^{a}B	3^{b}C
	40	4^{b}C	4^{b}C	5^{b}C	5^{c}C
12	-18	2^{a}A	3^{a}B	2^{a}A	1^{a}A
	5	3^{b}B	2^{b}A	3^{b}B	2^{b}B
	25	4^{c}B	2^{b}A	3^{b}B	3^{c}C
	40	5^{d}D	4^{c}C	6^{c}D	6^{d}D
15	5	3^{a}B	2^{a}A	3^{a}B	3^{a}C
	25	4^{b}B	2^{a}A	4^{b}C	3^{a}C
	40	5^{c}D	5^{b}D	6^{c}D	6^{b}D
18	5	3^{a}B	2^{a}A	3^{a}B	3^{a}C
	25	4^{b}B	2^{a}A	4^{b}C	3^{a}C
	40	5^{b}D	5^{b}D	6^{c}D	6^{b}D
21	5	3^{a}B	3^{a}B	3^{a}B	3^{a}C
	25	4^{b}B	3^{a}B	4^{b}C	3^{a}C
	40	5^{c}D	6^{b}E	7^{c}E	7^{b}E
24	-18	5^{a}B	4^{a}C	4^{a}B	3^{a}B
	5	3^{b}B	3^{b}B	4^{a}C	3^{a}C
	25	4^{c}B	3^{b}B	5^{b}D	4^{b}D
	40	5^{d}D	6^{c}E	7^{c}E	7^{c}E

* The means within a column (the difference between the storage temperature) followed by different superscript letters differ ($P < 0.05$). The means within a column (the difference between the storage period) followed by different capital letters differ ($P < 0.05$).

** Seven-point scales (1-excellent, 4-good, 7-unacceptable) were used.

*** A seven-point scale (1-negligible, 4-medium, 7-excessive) was used.

Table S6. Result of the amino acids content (g/kg) and the essential amino acids index (%) during a 24-month period at four different temperatures (-18°C; 5°C; 25°C and 40°C) in instant goulash soup. The results are expressed as means (n = 27); relative standard deviations ranged in the narrow range of 3.91–6.24%. *

Storage time (months)	Storage temperature (°C)	THR **		VAL		ILE		LEU		PHE		LYS		MET		ASP		SER	
0	-	3.6	A	4.1	A	5.7	A	8.0	A	3.7	A	6.5	A	2.5	A	13.8	A	3.3	A
12	– 18	3.5	^{a}A	4.0	^{a}A	5.7	^{a}A	7.9	^{a}A	3.5	^{a}A	6.3	^{a}B	2.4	^{a}B	13.2	^{a}A	3.2	^{a}A
	5	3.3	^{b}B	4.1	^{b}A	5.5	^{b}A	7.8	^{b}A	3.6	^{b}A	6.3	^{b}B	2.3	^{b}B	13.1	^{b}A	3.1	^{a}B
	25	3.1	^{c}B	3.9	^{c}B	5.4	^{c}B	7.6	^{c}B	3.5	^{c}B	5.9	^{c}B	2.2	^{c}B	12.9	^{c}B	3.0	^{b}B
	40	3.0	^{d}B	3.6	^{d}B	5.2	^{d}B	7.3	^{d}B	3.3	^{d}B	5.9	^{d}B	2.2	^{d}B	12.1	^{d}B	2.9	^{c}B
24	– 18	3.4	^{a}B	4.0	^{a}A	5.6	^{a}A	7.8	^{a}A	3.7	^{a}A	6.4	^{a}B	2.4	^{a}B	13.3	^{a}A	3.1	^{a}B
	5	3.1	^{b}C	3.9	^{b}B	5.5	^{a}A	7.7	^{b}B	3.5	^{b}B	5.9	^{b}C	2.2	^{b}C	13.1	^{b}B	2.9	^{b}C
	25	3.0	^{b}C	3.8	^{c}B	5.2	^{b}B	7.4	^{c}B	3.3	^{c}B	5.6	^{b}C	2.2	^{b}C	12.9	^{c}B	2.8	^{c}C
	40	2.8	^{c}C	3.5	^{d}B	5.1	^{c}B	7.1	^{d}B	3.2	^{d}B	5.4	^{c}C	2.1	^{c}C	11.3	^{d}C	2.7	^{d}C

Table S6. Continue

Storage time (months)	Storage temperature (°C)	GLU		PRO		GLY		ALA		TYR		HIS		ARG		CYS		Essential amino acids content	Total amino acids	EAAI
0	-	27.9	A	5.7	A	5.3	A	5.4	A	3.5	A	2.3	A	8.0	A	2.8	A	34.1	112.1	61.4
12	– 18	27.2	^{a}A	5.6	^{a}A	5.3	^{a}A	5.4	^{a}A	3.4	^{a}A	2.2	^{a}A	7.9	^{a}A	2.9	^{a}B	33.3	109.7	59.9
	5	27.0	^{b}A	5.7	^{b}A	5.2	^{b}A	5.3	^{b}A	3.3	^{b}B	2.2	^{b}A	7.8	^{b}A	2.7	^{b}B	33.0	108.5	59.1
	25	26.9	^{c}B	5.5	^{c}B	5.0	^{c}B	5.1	^{c}B	3.2	^{c}B	2.2	^{c}B	7.6	^{c}B	2.7	^{c}B	31.7	106.0	56.8
	40	25.8	^{d}B	5.2	^{d}B	4.7	^{d}B	5.0	^{d}B	3.1	^{d}B	2.0	^{d}B	7.3	^{d}B	2.6	^{d}B	30.4	100.6	54.2
24	– 18	27.2	^{a}A	5.6	^{a}A	5.2	^{a}A	5.4	^{a}A	3.3	^{a}B	2.2	^{a}A	7.8	^{a}A	2.8	^{a}B	33.3	109.2	59.8
	5	26.8	^{b}B	5.5	^{b}B	5.1	^{b}B	5.3	^{b}B	3.1	^{b}C	2.2	^{b}B	7.7	^{b}B	2.6	^{b}C	32.0	106.3	57.1
	25	25.8	^{c}B	5.2	^{c}B	4.9	^{c}B	5.0	^{c}B	2.9	^{b}C	2.0	^{c}B	7.3	^{c}B	2.5	^{b}C	30.5	101.4	54.6
	40	25.3	^{d}B	5.0	^{d}B	4.6	^{d}B	4.9	^{d}B	2.8	^{c}C	1.9	^{d}B	6.8	^{d}B	2.4	^{c}C	29.2	95.1	52.0

* The means within a column (the difference between the storage temperature at constant storage time) followed by different superscript letters differ ($P < 0.05$). The means within a column (the difference between the storage period at the same temperature) followed by different capital letters differ ($P < 0.05$). All samples were evaluated separately.

** THR – threonine, VAL – valine, ILE – isoleucine, LEU – leucine, PHE – phenylalanine, LYS – lysine, MET – methionine, ASP – asparagic acid and asparagine which is converted to asparagic acid during acid hydrolysis, SER – serine, GLU – glutamic acid and glutamine which is converted to glutamic acid during acid hydrolysis, PRO – proline, GLY – glycine, ALA – alanine, TYR – tyrosine, HIS – histidine, ARG – arginine, CYS – cysteine.

Table S7. Result of the amino acids content (g/kg) and the essential amino acids index (%) during a 24-month period at four different temperatures (-18°C; 5°C; 25°C and 40°C) in Szeged goulash. The results are expressed as means (n = 27); relative standard deviations ranged in the narrow range of 3.91–6.24%. *

Storage time (months)	Storage temperature (°C)	THR **		VAL		ILE		LEU		PHE		LYS		MET	ASP		SER	
0	-	1.6	A	2.0	A	1.5	A	3.6	A	2.0	A	3.5	A	0.8	4.4	A	1.4	A
12	– 18	1.6	^{a}A	2.0	^{a}A	1.5	^{a}A	3.6	^{a}A	2.0	^{a}A	3.3	^{a}B	0.7	4.3	^{a}A	1.4	^{a}A
	5	1.5	^{b}B	2.0	^{b}A	1.5	^{b}A	3.5	^{b}A	2.0	^{b}A	3.3	^{b}B	0.7	4.4	^{b}A	1.3	^{b}B
	25	1.5	^{c}B	2.0	^{c}B	1.5	^{c}B	3.5	^{c}B	1.9	^{c}B	3.2	^{c}B	0.7	4.2	^{c}B	1.3	^{c}B
	40	1.4	^{d}B	1.9	^{d}B	1.4	^{d}B	3.3	^{d}B	1.8	^{d}B	3.1	^{d}B	0.7	4.0	^{d}B	1.2	^{d}B
24	– 18	1.5	^{a}B	2.0	^{a}A	1.5	^{a}A	3.6	^{a}A	2.0	^{a}A	3.4	^{a}B	0.7	4.3	^{a}A	1.4	^{a}B
	5	1.4	^{b}C	2.0	^{b}B	1.5	^{b}B	3.5	^{b}B	2.0	^{b}B	3.2	^{b}C	0.7	4.2	^{b}B	1.3	^{b}C
	25	1.3	^{b}C	1.8	^{c}B	1.4	^{c}B	3.3	^{c}B	1.8	^{c}B	3.1	^{b}C	0.7	4.0	^{c}B	1.2	^{b}C
	40	1.3	^{c}C	1.8	^{d}B	1.3	^{d}B	3.1	^{d}B	1.7	^{d}B	2.9	^{c}C	0.6	3.7	^{d}B	1.1	^{c}C

Table S7. Continue

Storage time (months)	Storage temperature (°C)	GLU		PRO		GLY		ALA		TYR		HIS		ARG		CYS		Essential amino acids content	Total amino acids	EAAI
0	-	8.6	A	3.0	A	2.7	A	2.7	A	1.6	A	1.7	A	2.6	A	0.4	A	15.1	44.1	76.4
12	– 18	8.2	^{a}A	2.9	^{a}A	2.7	^{a}A	2.6	^{a}A	1.6	^{a}A	1.7	^{a}A	2.5	^{a}A	0.4	^{a}B	14.7	43.0	74.9
	5	8.4	^{b}A	2.9	^{b}A	2.7	^{b}A	2.5	^{b}A	1.5	^{b}B	1.7	^{b}A	2.5	^{b}A	0.4	^{b}B	14.5	42.7	73.4
	25	8.1	^{c}B	2.8	^{c}B	2.6	^{c}B	2.5	^{c}B	1.5	^{c}B	1.6	^{c}B	2.4	^{c}B	0.4	^{c}B	14.2	41.6	72.0
	40	7.7	^{d}B	2.7	^{d}B	2.4	^{d}B	2.4	^{d}B	1.4	^{d}B	1.5	^{d}B	2.3	^{d}B	0.3	^{d}B	13.6	39.6	68.7
24	– 18	8.3	^{a}A	2.9	^{a}A	2.7	^{a}A	2.6	^{a}A	1.6	^{a}B	1.7	^{a}A	2.5	^{a}A	0.4	^{a}B	14.7	43.1	74.6
	5	8.2	^{b}B	2.8	^{b}B	2.6	^{b}B	2.5	^{b}B	1.4	^{b}C	1.6	^{b}B	2.4	^{b}B	0.3	^{b}C	14.2	41.7	71.9
	25	7.9	^{c}B	2.7	^{c}B	2.5	^{c}B	2.4	^{c}B	1.4	^{b}C	1.6	^{c}B	2.4	^{c}B	0.3	^{b}C	13.4	39.6	67.7
	40	7.5	^{d}B	2.5	^{d}B	2.3	^{d}B	2.2	^{d}B	1.3	^{c}C	1.5	^{d}B	2.2	^{d}B	0.3	^{c}C	12.6	37.3	64.0

* The means within a column (the difference between the storage temperature at constant storage time) followed by different superscript letters differ ($P < 0.05$). The means within a column (the difference between the storage period at the same temperature) followed by different capital letters differ ($P < 0.05$). All samples were evaluated separately.

** THR – threonine, VAL – valine, ILE – isoleucine, LEU – leucine, PHE – phenylalanine, LYS – lysine, MET – methionine, ASP – asparagic acid and asparagine which is converted to asparagic acid during acid hydrolysis, SER – serine, GLU – glutamic acid and glutamine which is converted to glutamic acid during acid hydrolysis, PRO – proline, GLY – glycine, ALA – alanine, TYR – tyrosine, HIS – histidine, ARG – arginine, CYS – cysteine.

Table S8. Result of the amino acids content (g/kg) and the essential amino acids index (%) during a 24-month period at four different temperatures (-18°C; 5°C; 25°C and 40°C) in canned chicken meat. The results are expressed as means (n = 27); relative standard deviations ranged in the narrow range of 4,06–5,74%. *

Storage time (months)	Storage temperature (°C)	THR **		VAL		ILE		LEU		PHE		LYS		MET		ASP		SER	
0	-	9.4	A	8.7	A	7.4	A	13.3	A	6.1	A	14.1	A	3.6	A	14.0	A	6.2	A
12	– 18	9.3	^{a}A	8.7	^{a}A	7.3	^{a}A	13.0	^{a}A	6.1	^{a}A	13.4	^{a}B	3.5	^{a}B	13.8	^{a}A	6.0	^{a}A
	5	9.1	^{b}B	8.6	^{b}A	7.2	^{b}A	12.8	^{b}A	6.0	^{b}A	13.4	^{b}B	3.4	^{b}B	13.5	^{b}A	5.9	^{b}B
	25	8.7	^{c}B	8.4	^{c}B	7.2	^{c}B	12.8	^{c}B	5.8	^{c}B	12.8	^{c}B	3.3	^{c}B	13.4	^{c}B	5.6	^{c}B
	40	8.4	^{d}B	8.0	^{d}B	6.7	^{d}B	11.8	^{d}B	5.6	^{d}B	12.4	^{d}B	3.2	^{d}B	12.7	^{d}B	5.4	^{d}B
24	– 18	9.2	^{a}B	8.6	^{a}A	7.4	^{a}A	12.9	^{a}A	6.1	^{a}A	13.8	^{a}B	3.4	^{a}B	13.7	^{a}A	6.0	^{a}B
	5	8.5	^{b}C	8.4	^{b}B	7.1	^{b}B	12.8	^{b}B	6.0	^{b}B	12.5	^{b}C	3.2	^{b}C	13.5	^{b}B	5.6	^{b}C
	25	8.1	^{b}C	8.1	^{c}B	6.8	^{c}B	12.1	^{c}B	5.5	^{c}B	12.0	^{b}C	3.1	^{b}C	12.7	^{c}B	5.2	^{b}C
	40	7.8	^{c}C	7.4	^{d}B	6.5	^{d}B	11.6	^{d}B	5.3	^{d}B	11.7	^{c}C	2.9	^{c}C	12.4	^{d}B	5.1	^{c}C

Table S8. Continue

Storage time (months)	Storage temperature (°C)	GLU		PRO		GLY		ALA		TYR		HIS		ARG		CYS		Essential amino acids content	Total amino acids	EAAI
0	-	26.4	A	9.9	A	11.3	A	9.1	A	5.6	A	4.6	A	13.4	A	1.6	A	62.7	164.7	86.4
12	– 18	26.0	^{a}A	9.8	^{a}A	11.0	^{a}A	9.0	^{a}A	5.5	^{a}A	4.6	^{a}A	13.3	^{a}A	1.6	^{a}B	61.3	161.8	84.8
	5	25.5	^{b}A	9.6	^{b}A	10.8	^{b}A	8.8	^{b}A	5.3	^{b}B	4.6	^{b}A	13.1	^{b}A	1.5	^{b}B	60.4	159.0	83.2
	25	25.3	^{c}B	9.2	^{c}B	10.6	^{c}B	8.5	^{c}B	5.1	^{c}B	4.5	^{c}B	12.8	^{c}B	1.5	^{c}B	58.9	155.5	81.1
	40	23.8	^{d}B	8.8	^{d}B	10.2	^{d}B	8.1	^{d}B	4.8	^{d}B	4.3	^{d}B	12.0	^{d}B	1.4	^{d}B	56.1	147.5	77.4
24	– 18	25.9	^{a}A	9.7	^{a}A	10.8	^{a}A	8.8	^{a}A	5.4	^{a}B	4.5	^{a}A	13.2	^{a}A	1.6	^{a}B	61.4	161.0	84.6
	5	25.0	^{b}B	9.5	^{b}B	10.7	^{b}B	8.7	^{b}B	5.0	^{b}C	4.5	^{b}B	12.9	^{b}B	1.4	^{b}C	58.5	155.3	80.5
	25	24.3	^{c}B	8.9	^{c}B	10.1	^{c}B	8.2	^{c}B	4.8	^{b}C	4.3	^{c}B	12.3	^{c}B	1.4	^{b}C	55.8	147.8	76.8
	40	22.3	^{d}B	8.5	^{d}B	9.9	^{d}B	7.6	^{d}B	4.5	^{c}C	4.0	^{d}B	11.8	^{d}B	1.3	^{c}C	53.3	140.8	73.3

* The means within a column (the difference between the storage temperature at constant storage time) followed by different superscript letters differ ($P < 0.05$). The means within a column (the difference between the storage period at the same temperature) followed by different capital letters differ ($P < 0.05$). All samples were evaluated separately.

** THR – threonine, VAL – valine, ILE – isoleucine, LEU – leucine, PHE – phenylalanine, LYS – lysine, MET – methionine, ASP – asparagic acid and asparagine which is converted to asparagic acid during acid hydrolysis, SER – serine, GLU – glutamic acid and glutamine which is converted to glutamic acid during acid hydrolysis, PRO – proline, GLY – glycine, ALA – alanine, TYR – tyrosine, HIS – histidine, ARG – arginine, CYS – cysteine.

Table S9. Result of the amino acids content (g/kg) and the essential amino acids index (%) during a 24-month period at four different temperatures (-18°C; 5°C; 25°C and 40°C) in pork pâté. The results are expressed as means (n = 27); relative standard deviations ranged in the narrow range of 3.02–6,15%. *

Storage time (months)	Storage temperature (°C)	THR **		VAL		ILE		LEU		PHE		LYS		MET		ASP		SER	
0	-	8.0	A	13.5	A	8.6	A	17.6	A	11.8	A	18.9	A	6.8	A	20.7	A	9.9	A
12	– 18	7.8	^{a}A	13.4	^{a}A	8.5	^{a}A	17.8	^{a}A	11.5	^{a}A	18.4	^{a}B	6.5	^{a}B	20.8	^{a}A	9.6	^{a}A
	5	7.7	^{b}B	13.1	^{b}A	8.3	^{b}A	17.4	^{b}A	11.3	^{b}A	18.0	^{b}B	6.5	^{b}B	19.9	^{b}A	9.4	^{b}B
	25	7.3	^{c}B	13.0	^{c}B	8.0	^{c}B	16.8	^{c}B	11.1	^{c}B	17.8	^{c}B	6.3	^{c}B	19.9	^{c}B	9.0	^{c}B
	40	7.1	^{d}B	12.3	^{d}B	7.6	^{d}B	15.9	^{d}B	10.4	^{d}B	17.2	^{d}B	6.0	^{d}B	18.8	^{d}B	8.8	^{d}B
24	– 18	7.6	^{a}B	13.4	^{a}A	8.6	^{a}A	17.5	^{a}A	11.5	^{a}A	18.5	^{a}B	6.6	^{a}B	20.5	^{a}A	9.3	^{a}B
	5	7.2	^{b}C	13.0	^{b}B	8.1	^{b}B	17.2	^{b}B	11.2	^{b}B	17.2	^{b}C	6.1	^{b}C	20.0	^{b}B	8.9	^{b}C
	25	6.8	^{b}C	12.5	^{c}B	7.9	^{c}B	16.0	^{c}B	10.6	^{c}B	16.8	^{b}C	5.9	^{b}C	19.3	^{c}B	8.3	^{b}C
	40	6.5	^{c}C	11.9	^{d}B	7.4	^{d}B	15.1	^{d}B	10.0	^{d}B	16.0	^{c}C	5.7	^{c}C	18.6	^{d}B	8.1	^{c}C

Table S9. Continue

Storage time (months)	Storage temperature (°C)	GLU		PRO		GLY		ALA		TYR		HIS		ARG		CYS		Essential amino acids content	Total amino acids	EAAI
0	-	38.4	A	26.4	A	37.4	A	16.7	A	6.0	A	9.2	A	20.3	A	4.1	A	85.2	274.3	70.2
12	– 18	38.7	^{a}A	26.1	^{a}A	36.7	^{a}A	16.1	^{a}A	5.9	^{a}A	9.2	^{a}A	20.1	^{a}A	4.0	^{a}B	83.8	270.9	68.9
	5	38.3	^{b}A	25.6	^{b}A	36.5	^{b}A	16.3	^{b}A	5.8	^{b}B	9.0	^{b}A	19.6	^{b}A	3.8	^{b}B	82.3	266.5	67.7
	25	37.2	^{c}B	25.0	^{c}B	35.4	^{c}B	15.6	^{c}B	5.4	^{c}B	8.9	^{c}B	19.7	^{c}B	3.7	^{c}B	80.3	260.1	65.9
	40	35.2	^{d}B	24.1	^{d}B	33.4	^{d}B	14.9	^{d}B	5.1	^{d}B	8.3	^{d}B	18.3	^{d}B	3.6	^{d}B	76.7	247.3	62.9
24	– 18	38.8	^{a}A	25.9	^{a}A	36.6	^{a}A	16.1	^{a}A	5.9	^{a}B	9.1	^{a}A	20.2	^{a}A	4.0	^{a}B	83.6	270.1	68.8
	5	37.9	^{b}B	25.1	^{b}B	35.8	^{b}B	15.7	^{b}B	5.3	^{b}C	8.7	^{b}B	19.4	^{b}B	3.6	^{b}C	80.0	260.4	65.6
	25	35.6	^{c}B	24.2	^{c}B	33.9	^{c}B	14.9	^{c}B	5.1	^{b}C	8.3	^{c}B	18.4	^{c}B	3.6	^{b}C	76.5	248.1	62.7
	40	34.9	^{d}B	23.8	^{d}B	33.1	^{d}B	14.6	^{d}B	4.8	^{c}C	7.8	^{d}B	17.3	^{d}B	3.3	^{c}C	72.6	238.9	59.6

* The means within a column (the difference between the storage temperature at constant storage time) followed by different superscript letters differ (P < 0.05). The means within a column (the difference between the storage period at the same temperature) followed by different capital letters differ (P < 0.05). All samples were evaluated separately.

** THR – threonine, VAL – valine, ILE – isoleucine, LEU – leucine, PHE – phenylalanine, LYS – lysine, MET – methionine, ASP – asparagic acid and asparagine which is converted to asparagic acid during acid hydrolysis, SER – serine, GLU – glutamic acid and glutamine which is converted to glutamic acid during acid hydrolysis, PRO – proline, GLY – glycine, ALA – alanine, TYR – tyrosine, HIS – histidine, ARG – arginine, CYS – cysteine.

Table S10. Result of the amino acids content (g/kg) and the essential amino acids index (%) during a 24-month period at four different temperatures (-18°C; 5°C; 25°C and 40°C) in canned tuna fish. The results are expressed as means (n = 27); relative standard deviations ranged in the narrow range of 5,04–6,92%. *

Storage time (months)	Storage temperature (°C)	THR **		VAL		ILE		LEU		PHE		LYS		MET		ASP		SER	
0	-	7.2	A	9.5	A	8.4	A	13.4	A	8.1	A	15.2	A	6.5	A	17.0	A	6.1	A
12	– 18	7.1	^{a}A	9.4	^{a}A	8.1	^{a}A	13.5	^{a}A	8.0	^{a}A	15.3	^{a}B	6.5	^{a}B	16.7	^{a}A	6.0	^{a}A
	5	7.0	^{b}B	9.2	^{b}A	8.2	^{b}A	13.4	^{b}A	7.8	^{b}A	14.8	^{b}B	6.2	^{b}B	16.4	^{b}A	5.8	^{b}B
	25	6.6	^{c}B	9.0	^{c}B	8.0	^{c}B	12.9	^{c}B	7.8	^{c}B	14.1	^{c}B	6.1	^{c}B	16.1	^{c}B	5.6	^{c}B
	40	6.6	^{d}B	8.7	^{d}B	7.6	^{d}B	12.2	^{d}B	7.2	^{d}B	13.7	^{d}B	5.8	^{d}B	15.2	^{d}B	5.4	^{d}B
24	– 18	7.1	^{a}B	9.2	^{a}A	8.2	^{a}A	13.4	^{a}A	7.7	^{a}A	14.8	^{a}B	6.4	^{a}B	16.8	^{a}A	5.9	^{a}B
	5	6.5	^{b}C	9.2	^{b}B	8.1	^{b}B	13.1	^{b}B	7.7	^{b}B	13.6	^{b}C	5.9	^{b}C	16.5	^{b}B	5.5	^{b}C
	25	6.2	^{b}C	8.6	^{c}B	7.5	^{c}B	12.3	^{c}B	7.2	^{c}B	13.2	^{b}C	5.5	^{b}C	15.3	^{c}B	5.2	^{b}C
	40	5.9	^{c}C	8.3	^{d}B	7.2	^{d}B	11.9	^{d}B	6.9	^{d}B	12.9	^{c}C	5.4	^{c}C	14.9	^{d}B	4.9	^{c}C

Table S10. Continue

Storage time (months)	Storage temperature (°C)	GLU		PRO		GLY		ALA		TYR		HIS		ARG		CYS		Essential amino acids content	Total amino acids	EAAI
0	-	21.1	A	5.5	A	7.8	A	10.1	A	5.0	A	14.4	A	11.5	A	2.9	A	68.3	169.9	91.7
12	– 18	20.8	^{a}A	5.6	^{a}A	7.6	^{a}A	10.1	^{a}A	4.9	^{a}A	14.0	^{a}A	11.1	^{a}A	2.8	^{a}B	67.8	167.4	90.8
	5	20.4	^{b}A	5.4	^{b}A	7.5	^{b}A	9.8	^{b}A	4.7	^{b}B	13.9	^{b}A	11.1	^{b}A	2.8	^{b}B	66.6	164.3	89.1
	25	19.7	^{c}B	5.2	^{c}B	7.3	^{c}B	9.7	^{c}B	4.6	^{c}B	13.4	^{c}B	10.9	^{c}B	2.7	^{c}B	64.5	159.8	86.4
	40	18.9	^{d}B	5.0	^{d}B	7.0	^{d}B	8.9	^{d}B	4.4	^{d}B	12.6	^{d}B	10.2	^{d}B	2.5	^{d}B	61.7	152.0	82.8
24	– 18	20.5	^{a}A	5.5	^{a}A	7.6	^{a}A	10.0	^{a}A	4.8	^{a}B	13.7	^{a}A	11.0	^{a}A	2.8	^{a}B	66.9	165.5	89.8
	5	20.2	^{b}B	5.4	^{b}B	7.4	^{b}B	9.8	^{b}B	4.5	^{b}C	13.8	^{b}B	10.9	^{b}B	2.6	^{b}C	64.0	160.7	85.9
	25	18.9	^{c}B	5.0	^{c}B	7.0	^{c}B	9.2	^{c}B	4.3	^{b}C	13.1	^{c}B	10.4	^{c}B	2.5	^{b}C	60.6	151.5	81.0
	40	18.6	^{d}B	4.8	^{d}B	6.7	^{d}B	8.7	^{d}B	4.0	^{c}C	12.4	^{d}B	9.9	^{d}B	2.2	^{c}C	58.5	145.5	78.2

* The means within a column (the difference between the storage temperature at constant storage time) followed by different superscript letters differ ($P < 0.05$). The means within a column (the difference between the storage period at the same temperature) followed by different capital letters differ ($P < 0.05$). All samples were evaluated separately.

** THR – threonine, VAL – valine, ILE – isoleucine, LEU – leucine, PHE – phenylalanine, LYS – lysine, MET – methionine, ASP – asparagic acid and asparagine which is converted to asparagic acid during acid hydrolysis, SER – serine, GLU – glutamic acid and glutamine which is converted to glutamic acid during acid hydrolysis, PRO – proline, GLY – glycine, ALA – alanine, TYR – tyrosine, HIS – histidine, ARG – arginine, CYS – cysteine.
